# Associations between fecal short-chain fatty acids and sleep continuity in older adults with insomnia symptoms

**DOI:** 10.1038/s41598-021-83389-5

**Published:** 2021-02-18

**Authors:** Faiga Magzal, Carmel Even, Iris Haimov, Maayan Agmon, Kfir Asraf, Tamar Shochat, Snait Tamir

**Affiliations:** 1 Laboratory of Human Health and Nutrition Sciences, MIGAL-Galilee Research Institute, Kiryat Shmona, Israel; 2 Tel Hai College, Upper Galilee, Israel; 3 The Max Stern Yezreel Valley College, Emek Yezreel, Israel; 4 University of Haifa, Haifa, Israel

**Keywords:** Chemical biology, Microbiology, Neuroscience, Diseases

## Abstract

Insomnia is a disorder characterized by difficulty falling asleep and poor sleep continuity and is associated with increased risks for physical and cognitive decline. Insomnia with short sleep duration is considered the most biologically severe phenotype of the disorder. Evidence suggests that short-chain fatty acids (SCFAs), the main byproducts of fiber fermentation in the gut, may affect sleep via gut–brain communications. This study explores associations between SCFAs and sleep continuity and compares SCFA concentrations in short vs. normal sleep insomnia phenotypes in older adults. Fifty-nine participants with insomnia symptoms (≥ 65 years), completed 2 weeks of objective sleep monitoring (actigraphy), and were divided into short and normal sleep duration phenotypes via cluster analysis. Sleep measures included total sleep time (TST), sleep onset latency (SOL), sleep efficiency (SE), and wake after sleep onset (WASO). Stool samples were collected and fecal SCFA concentrations were determined by gas-chromatography-mass-spectrometry (GCMS). Higher concentrations of acetate, butyrate, and propionate, and total SCFAs, were associated with lower SE and longer SOL after controlling for Body Mass Index (BMI). Concentrations were higher in the short sleep duration phenotype. Age, BMI, TST, and SOL explained 40.7% of the variance in total SCFAs. Findings contribute to understanding pathways along the gut–brain axis and may lead to the use of SCFAs as biomarkers of insomnia phenotypes.

## Introduction

Insomnia is a common chronic health condition and the most common sleep disorder, with symptoms affecting around 50% of the adult population over 65 years old^1^. It is defined by the Diagnostic and Statistical Manual of Mental Disorders, 5th edition (DSM-5)^2^, as difficulty initiating sleep, difficulty maintaining sleep and/or early morning awakening, at least 3 times per week, lasting > 3 months, and is associated with an impairment of daytime functioning, e.g. impairment of attention, memory or mood^2^. In older adults, insomnia is particularly associated with nighttime symptoms (i.e., SOL and WASO) and hyperarousal^1^. Its persistence over time is detrimental to physical and psychological wellbeing and health-related quality of life^2–4^. Insomnia is positively associated with systemic inflammation^5^ and all-cause mortality^6^. Models proposed by Irwin^7^ (2019), demonstrate the complex associations between sleep disturbances, operationalized as short sleep duration and reduced sleep continuity, stress, and chronic inflammation. This sleep disturbance shifts the transcriptional profile towards increased inflammatory activity, increasing inflammatory markers (IL-6 and C-reactive protein and Tumor Necrosis Factor) and inflammatory transcriptional activity^7,8^ (Nuclear Factor-κB and Activator Protein 1). Yet the metabolic mechanisms underlying the relationship between insomnia and inflammation are still poorly understood.

Short-chain fatty acids (SCFAs) are produced by means of the fermentation of host-indigestible dietary fibers by specific bacteria in the human gut^9^, and are involved in numerous physiological functions such as gastrointestinal functionality, host metabolism, blood pressure regulation, neuroimmune function^10–13^, and gut–brain communication^14,15^. Acetate, propionate, and butyrate are the most abundant SCFAs present in the colon. They affect systemic inflammation mainly by regulating the secretion of interleukins and by inducing T regulatory cell differentiation^16^.

In human feces, higher SCFA concentrations are associated with increased gut permeability, metabolic dysregulation, and hypertension^17^, all of which are increased in older age^18–20^. Reduced absorption of circulating SCFAs has been proposed as an explanation for the increased cardiometabolic risk associated with a higher concentration of fecal SCFAs^17^. Indeed, an inverse association has been shown between acetate concentrations in feces and colonic acetate absorption^21^.

SCFAs exert widespread effects on neurological and behavioral processes via the gut–brain axis. By crossing the blood–brain barrier (BBB), they have been shown to increase neurogenesis, improve neuronal homeostasis and function and contribute to serotonin biosynthesis^22^. While associations between serotonin and depression have been widely acknowledged for decades^23,24^, a large body of literature has established the role of serotonin in sleep–wake regulation^25,26^.

Furthermore, recent evidence suggests that SCFAs may function as mediators linking gut bacteria and sleep mechanisms in the brain^27,28^, but findings are scarce and inconsistent. Acetate has been shown to be involved in appetite regulation by altering levels of inhibitory GABA and excitatory glutamate and glutamine neurotransmitters in the hypothalamus^29^. Reduced GABA levels^30–32^ and elevated glutamate and glutamine^33^ are implicated in increased hyperarousal that characterizes individuals with insomnia. In addition, there is some evidence to suggest that reduced GABA levels are correlated with shorter sleep duration^30^. Also, butyrate treatment in vitro and in vivo has been shown to inhibit depression-like behavior in rodents^34^. One study showed that oral administration or direct intraportal injection of butyrate and tributyrin, a butyrate-yielding pro-drug, elicited an almost 50% increase in non-rapid-eye movement in young mice for 4 h after treatment^27^. A recent animal study found increased concentrations of butyrate in mice demonstrating increased sleep duration and reduced sleep onset latency after receiving high-dose GABA fermented milk^35^. In human infants, increased fecal propionate, but not acetate or butyrate, was associated with longer sleep duration^28^. However, in young fruit flies, dietary administration of medium- and long-chain fatty acids, but not short-chain fatty acids, increased sleep duration in a dose-dependent manner^36^. These associations have yet to be observed in older human adults with insomnia, which represents a chronic condition of disturbed and inadequate sleep.

Growing evidence supports a distinction between two phenotypes of insomnia based on objective sleep duration: short and normal^37^. Insomnia with objective short sleep duration, typically defined as ≤ 6 h^38,39^, is the most biologically severe phenotype and is associated with physiological hyperarousal and increased risk of cardiometabolic morbidity and mortality^38,40,41^. In contrast, the normal sleep duration phenotype (typically defined as > 6 h) is associated with an overall lower risk of cardiometabolic outcomes^38,39^. As insomnia with short sleep duration is considered biologically based^39^, the potential underlying biological mechanisms of these two insomnia prototypes are likely to be different.

Our research focuses on older adults as a vantage point in our aim to understand relationships between two distinct physiological functions that typically show accelerated decline throughout the aging process; i.e., sleep (represented by the high prevalence of insomnia symptoms in older adults) and metabolism (represented by declined absorption of SCFAs, which is associated with age-related cardiometabolic disturbances). Given the importance of SCFAs in systemic inflammation and brain function, and given the evidence that they promote sleep, we aimed to investigate associations between fecal SCFA concentrations and sleep duration and continuity measures assessed by actigraphy in older adults with insomnia symptoms, comparing short and normal sleep insomnia phenotypes. As fecal SCFA concentrations indicate low absorption and negative metabolic outcomes, we hypothesized that increased fecal SCFA concentrations would be associated with longer sleep onset latency, poorer sleep continuity (i.e., increased wake after sleep onset, and lower sleep efficiency) and with the short sleep duration insomnia phenotype.

## Materials and methods

The institutional review board (IRB) of the Faculty of Social Welfare and Health Sciences at the University of Haifa approved this study and all its methods, conforming to relevant guidelines and regulations (approval number 026/17). All study participants signed informed consent.

### Participants

Two hundred and twenty-four potential participants, ages 65 and older, responded to advertisements and activities in community centers for older adults, calling for volunteers with an insomnia complaint and/or sleep dissatisfaction lasting several months at least. Eighty participants were eligible. The presence of insomnia symptoms was based on self-report and supplemented by 2 weeks of actigraphic recordings. Participants underwent a short clinical interview conducted by a trained interviewer by telephone, during which they were asked about medical condition(s), medication(s) or other substance use, and responded to questions about specific nighttime sleep problems (see questions below). Eligible participants reported that they experienced insomnia symptoms at least 3 nights per week, and on those nights reported > 30 min falling asleep, and/or > 30 min awakenings. Based on actigraphy, inclusion criteria for insomnia symptoms were comprised of the following commonly used benchmarks for poor sleep continuity:^42,43^ (a) sleep onset latency (SOL) or wake after sleep onset (WASO) of > 30 min; and (b) less than 85% sleep efficiency (SE, percentage of total sleep time after initial sleep onset out of total time in bed) for at least three out of seven nights each week. Exclusion criteria included any significant visual or hearing impairments; chronic pain; substantial and unstable medical, neurological, or psychiatric illness; alcohol or drug use; psychiatric medication use; sleep apnea syndrome (SAS); and periodic limb movement disorder during sleep (PLMD), based on self-report. Participants were tested on the Mini-Mental State Examination (MMSE)^44^, using a cutoff > 26 to exclude participants with cognitive impairment. All participants were Jewish and highly educated, 60% were married and 65% lived with their spouse.

All study participants were scheduled a home visit during which registered dietitians administered questionnaires on demographics, anthropometrics and nutritional data and medical history and collected stool samples. BMI was calculated based on participants weight and height measurements taken by the dietitian and calculated using the equation: Weight/(Height)^2^. SCFA analysis was performed on fifty-nine participants (11 males, 48 females) that were included in the present study.

### Sleep assessment measurements

#### Clinical interview

Specific sleep complaints included the following items: (1) *How many times a week do you experience insomnia, i.e., have difficulty falling asleep or difficulty staying asleep *(*waking up at night and have difficulty falling back to sleep*)? (2)* On nights with insomnia: *(2a)* how long does it take you to fall asleep*? (2b) *how long are you awake due to nighttime awakenings*?

Two additional yes/no questions were asked regarding sleep apnea (“*Have you been diagnosed with sleep apnea*?”) and periodic limb movements disorder (“*have you been diagnosed with periodic limb movements disorder in sleep?*”).

### Actigraphy

Participants wore an Actiwatch (Phillips Respironics), activity wrist monitor for a 2-week period. Recordings were performed in one-minute epochs with the Actiware 6.0.9 algorithm. Sleep onset and offset were set to the first and last epoch of ten consecutive immobile minutes, respectively. To establish and mark the timing of rest intervals, participants were instructed (1) to press on the event marker, a built-in feature of the Actiwatch, at bedtime and at final awakening, and (2) to complete a sleep diary each morning upon awakening (on paper). Rest intervals were based primarily on event markers and were verified by sleep diary records. Participants wore an Actiwatch (Phillips Respironics) activity wrist monitor for a 2-week period. Derived measures included total sleep time (TST, minutes of sleep from intended bedtime to final wake time), sleep onset latency (SOL, minutes to fall asleep from bedtime), sleep efficiency (SE, percentage of total sleep time after initial sleep onset), and wake time after sleep onset (WASO, total wake minutes after sleep onset).

### Stool sampling

Participants were asked to collect a stool sample during the morning hours into a screw-capped collection container using a plastic holder and containing an RNase inhibitor solution (DNA/RNA Shield Fecal Collection Tube, Zymo Research, CA, USA). Stool specimens were taken to the laboratory and stored at − 20 °C until the time of analysis.

### SCFAs extraction and analysis

SCFAs were extracted and analyzed according to a modified version of the method described by Zhao et al.^45^. Briefly, each sample was thoroughly mixed using a vortex for 5 min. An aliquot of 0.5 ml of the mixed fecal solution was taken. Its pH was adjusted to 2–3 by adding 150 μl of orthophosphoric acid (16% v/v), after which it was kept at room temperature for 10 min with occasional shaking. The suspension was transferred into a polypropylene tube and centrifuged at 4 °C for 5 min at 10,000 rpm. The supernatant was transferred to a chromatographic vial for gas chromatography analyses, and 2-methyl-butyric-acid (#109959, Sigma-Aldrich, USA) was added to each vial in order to reach a final concentration of 0.001 M. 2-methyl-butyric-acid was added as an internal standard (IS) to correct for injection variability between samples and minor changes in the instrument response. All vials were stored at − 20 °C before GC analysis. The feces sediment was dried at 60 °C for 5–7 days, and its weight determined.

### Gas chromatography analysis

The GCMS system consisted of an Agilent 7890A (Agilent Technologies, Palo Alto, CA, USA), equipped with an automatic liquid sampler (MPS2) (Gerstel, Mulheim, Germany) and coupled to an Agilent 5975C mass selective detector. Results were acquired using the Chemstation software (Hewlett-Packard, Palo Alto, CA, USA). The column used was a fused-silica capillary column with a free fatty acid phase (DB-FFAP 122-3232, J&W Scientific, Agilent Technologies Inc., USA) of 0.25 mm × 30 m × 0.25 μm. We used Helium as the carrier gas at a flow rate of 60.453 mL/min. The initial oven temperature was 70 °C, held for 0.75 min, raised to 160 °C at 5 °C/min, raised to 230 °C at 20 °C/min, and held for 5.0 min. To avoid the contamination with nonvolatile fecal material, a glass liner with a glass wool plug at the lower end of the GC column. The injected sample volume for GC analysis was 1 μl, and the run time for each analysis was 27.25 min. The detector was operated in the selection ionization mode (SIM). Ion selection of the SCFAs was based on the retention time of standard compounds (WSFA-4, #47056, Sigma-Aldrich, USA).

### Statistical analysis

We divided participants into insomnia phenotypes by the SPSS K-means cluster test, using sleep efficiency and sleep duration derived from actigraphy as two distinguishing features of sleep. This method looks at variation in specific variables and creates 2 or more groups with maximal variance between them. Two clusters emerged, with no differences in sleep efficiency but significant differences in sleep duration. Based on differences in sleep duration, we labeled the first cluster “normal sleep duration” (normal group; TST = 7.33 ± 0.62 h, n = 36) and the second cluster “short sleep duration” (short group; TST = 5.64 ± 0.67 h, n = 23).

Mann–Whitney, Pearson's Chi-square and Fisher's exact tests were performed to determine differences in demographic and clinical features, sleep measures, and SCFA levels between clusters. Partial Spearman correlation tests, controlling for BMI (as BMI was significantly different between groups), were performed to detect associations between SCFA concentrations and actigraphy-derived sleep measures: TST, SOL, SE, and WASO. To test whether two correlations were significantly different between groups, we performed Fisher r-to-z transformations (https://www.psychometrica.de/correlation.html).

A multiple linear regression model was used to assess the associations between sleep measures and total SCFAs, controlling for age and BMI. All statistical tests were performed using SPSS software, version 26.

## Results

### Participant characteristics

Table 1 summarizes the characteristics of the combined sample and of both groups separately. No group differences were found for age, sex, education, marital status, living status, metabolic diseases or use of medications, daily consumption of dietary fiber and protein, or SOL and SE. BMI was significantly higher and TST and WASO were significantly shorter in the short group as compared to the normal group.

**Table 1:** Characteristics of the study population.

Parameter	Combined sample (n = 59)	Normal sleep duration (n = 36)	Short sleep duration (n = 23)	U (Z)/χ^2^ (df)	*P *(normal vs. short)
Age (years)	73.3 ± 4.8	73.7 ± 5.5	72.7 ± 3.4	384.5 (Z = − 0.10)	0.916
**Sex (%)**					
Female	81.4	80.6	82.6	^	> 0.999
Male	18.6	19.4	17.4		
Education (years)	16.09 ± 3.69	16.38 ± 4.37	15.66 ± 2.39	314 (Z = − 1.26)	0.206
**Marital status (%)**					
Married	61.4	57.1	68.2	0.69 (df = 1)	0.405
Other	38.6	42.9	31.8		
**Living status (%)**					
Lives alone	34.5	37.1	30.4	0.27 (df = 1)	0.599
Lives with a partner/s	65.5	62.9	69.6		
BMI (kg/m^2^)	29.6 ± 6.6	26.1 ± 4.0	32.6 ± 7.9	205.5 (Z = − 3.24)	***0.001***
**Dietary intake**					
Protein (g/day)	92.3 ± 31.0	98.4 ± 32.6	82.9 ± 26.4	199 (Z = − 1.61)	0.107
Dietary fiber (g/day)	38.2 ± 13.2	41.5 ± 14.7	33.1 ± 8.7	186 (Z = − 1.88)	0.059
**Metabolic diseases (%)**					
Hypertension	49.2	41.7	60.9	2.07 (df = 1)	0.150
Diabetes	22	16.7	30.4	1.54 (df = 1)	0.213
Hypercholesterolemia	42.4	50.0	30.4	2.20 (df = 1)	0.138
**Use of medications (%)**					
Sleep medications	14.6	16.7	11.1	^	0.696
Depression medications	12.2	12.9	11.1	^	> 0.999
Anti-cholinergic medication	19.1	16.7	23.5	^	0.704
**Sleep parameters**					
TST (h)	6.7 ± 1.0	7.32 ± 0.61	5.64 ± 0.66	0 (Z = − 6.43)	***<0.001***
SOL (min)	16.5 ± 12.3	16.8 ± 13.0	16.0 ± 11.4	407 (Z = − 0.10)	0.913
SE (%)	81.0 ± 8.2	81.9 ± 6.9	79.6 ± 9.9	374 (Z = − 0.62)	0.534
WASO (min)	52.9 ± 25.1	57.9 ± 25.7	45.1 ± 22.5	248 (Z = − 2.57)	***0.009***
**Short-chain fatty acids**					
Acetate	145.46 ± 64.18	127.59 ± 53.16	173.42 ± 70.89	261 (Z = − 2.37)	***0.017***
Propionate	27.22 ± 24.53	20.9 ± 16.05	37.1 ± 31.77	240.5 (Z = − 2.69)	***0.007***
Butyrate	40.06 ± 32.25	32.11 ± 24.28	52.5 ± 39.22	235 (Z = − 2.78)	***0.005***
Isobuyrate	1.06 ± 2.58	1.05 ± 2.75	1.09 ± 2.33	396.5 (Z = − 0.32)	0.743
Isovalerate	1.91 ± 1.87	1.8 ± 1.79	2.06 ± 2.03	386.5 (Z = − 0.42)	0.669
Valerate	3.2 ± 2.91	2.72 ± 2.57	3.96 ± 3.3	308 (Z = − 1.64)	0.099
Total SCFAs	218.93 ± 118.89	186 ± 90.21	270.17 ± 140.79	243 (Z = − 2.65)	***0.008***

Bold-italicized numbers are statistically significant results with p < 0.05. ^Based on Fisher's exact test.

Variables are presented as mean ± SD for the combined sample and for the normal and short groups separately. The p-values are from chi-square tests (for the parameters of age, living status, marital status, metabolic conditions, and medication use), and Mann–Whitney tests (for dietary intake parameters, sleep parameters, age, and BMI) between both groups. TST = total sleep time, SE = sleep efficiency, WASO = wake after sleep onset, and SOL = sleep onset latency.

**Figure 1:**
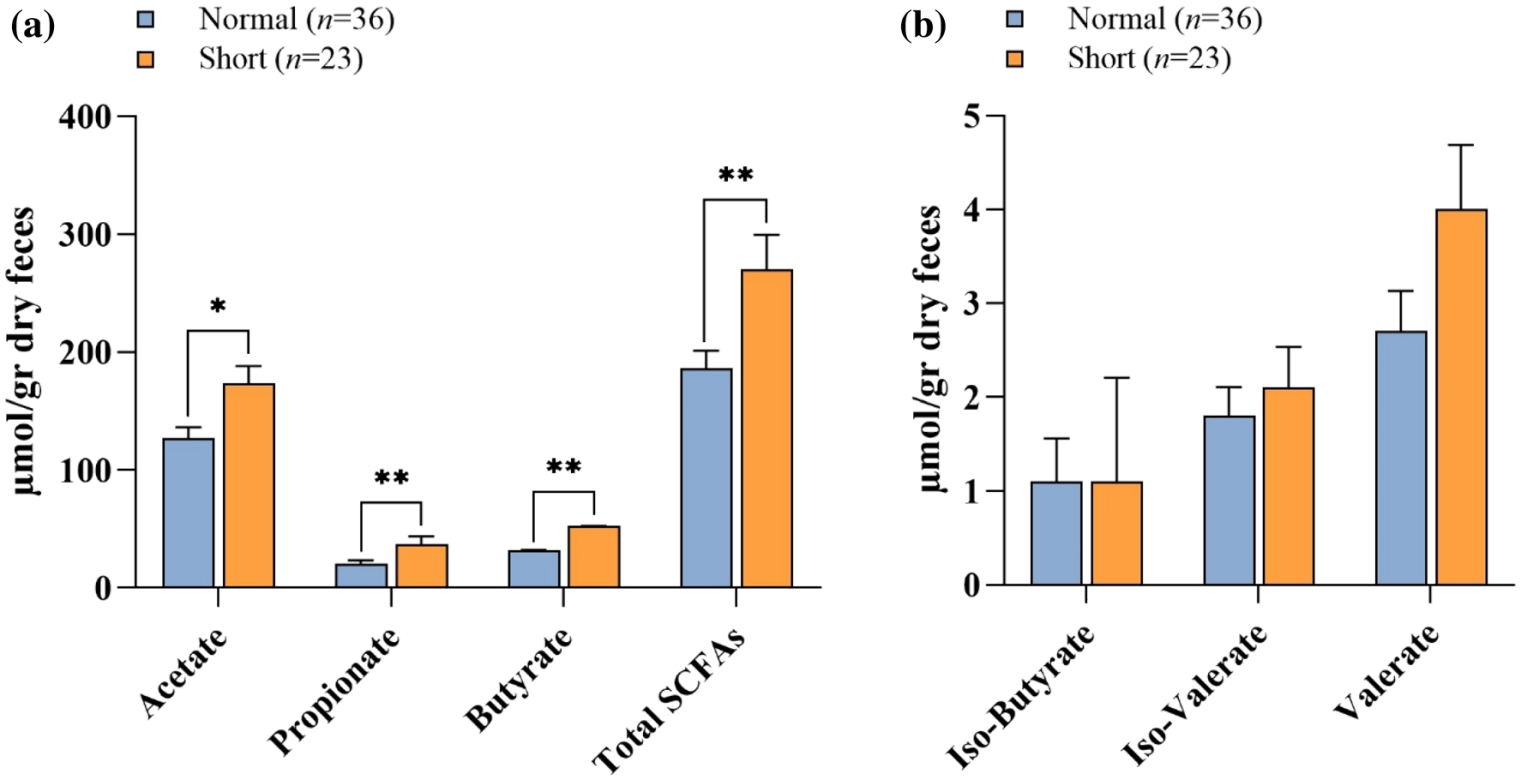
Differences in SCFA concentrations in the Normal and Short groups. The SCFAs measured are acetate, propionate, and butyrate (**a**), and iso-butyrate, iso-valerate, and valerate (**b**). Results are shown as mean ± SE. A Mann–Whitney test was used to compare differences between groups. *p < 0.05, **p < 0.01.

Levels of all SCFAs were determined for the entire sample and by group (Table 1 and Fig. 1). Acetate was the most abundant SCFA present, with an average of 145.5 ± 64.2 μmol per gram dry feces, followed by butyrate (40.1 ± 32.3 μmol per gram dry feces) and propionate (27.2 ± 24.5 μmol per gram dry feces). Concentrations of isobutyrate, isovalerate, and valerate were low, ranging from 0 to 13.6 μmol per gram of dry feces. Acetate, butyrate, propionate, and total SCFAs were significantly higher in the short group than in the normal group (Fig. 1a). Isobutyrate, isovalerate, and valerate were not significantly different between groups (Fig. 1b).

### Correlation results

Spearman coefficients and p-values for all significant and non-significant associations between SCFAs and sleep parameters, adjusted for the effects of BMI, are shown in Table 2. In the combined sample, higher acetate concentrations were associated with increased SOL and with decreased SE. In both short and normal insomnia groups, acetate was significantly associated with SOL. Acetate was significantly associated with SE in the short sleep duration group. No significant associations were found between acetate and WASO.

**Table 2:** Spearman's partial correlation coefficients and p-values (in parentheses) between SCFA concentrations and sleep measures in the Study Population (n = 59), Normal Sleep Duration participants (n = 36), and Short Sleep Duration participants (n = 23) adjusted for the effects of BMI.

	All participants (n = 59) Adjusted for BMI				Normal sleep duration participants (n = 36) Adjusted for BMI				Short sleep duration participants (n = 23) Adjusted for BMI			
	TST (h)	SOL	SE	WASO	TST	SOL	SE	WASO	TST	SOL	SE	WASO
Acetate	− 0.275 (0.037)	0.522 (***<0.001***)	− 0.511 (***< 0.001***)	0.142 (0.288)	− 0.121 (0.490)	0.524 (***0.001***)	− 0.402 (0.017)^†^	0.129 (0.459)	− 0.263 (0.237)	0.565 (***0.006***)	− 0.685 (***< 0.001***)^†^	0.352 (0.108)
Propionate	− 0.286 (0.029)	0.387 (***0.003***)	− 0.361 (***0.005***)	0.108 (0.419)	− 0.240 (0.164)	0.314 (0.066)^†^	− 0.154 (0.376)^†^	− 0.018 (0.917)	− 0.252 (0.258)	0.628 (***0.002***)^†^	− 0.744 (***< 0.001***)^†^	0.464 (*0.030*)
Butyrate	− 0.338 (***0.009***)	0.411 (***0.001***)	− 0.457 (***< 0.001***)	0.134 (0.315)	− 0.265 (0.124)	0.312 (0.068)	− 0.347 (0.041)^†^	0.143 (0.413)	− 0.302 (0.172)	0.564 (***0.006***)	− 0.653 (***< 0.001***)^†^	0.351 (0.109)
Valerate	− 0.162 (0.223)	0.117 (0.383)	− 0.165 (0.215)	0.06 (0.654)	− 0.228 (0.188)	0.095 (0.587)	− 0.101 (0.563)	− 0.075 (0.669)	− 0.053 (0.815)	0.164 (0.466)	− 0.268 (0.228)	0.242 (0.278)
Isobutyrate	− 0.011 (0.933)	0.133 (0.32)	− 0.209 (0.115)	0.195 (0.142)	0.082 (0.639)	0.158 (0.365)	− 0.181 (0.298)	0.207 (0.233)	0.031 (0.889)	0.101 (0.654)	− 0.294 (0.185)	0.390 (0.072)
Isovalerate	− 0.001 (0.995)	0.034 (0.803)	− 0.065 (0.626)	0.056 (0.676)	− 0.193 (0.268)	− 0.012 (0.946)	0.000 (0.999)	− 0.067 (0.702)	0.227 (0.309)	0.153 (0.496)	− 0.172 (0.444)	0.207 (0.356)
Total SCFAs	− 0.310 (0.018)	0.488 (***0.001***)	− 0.488 (***<0.001***)	0.123 (0.359)	− 0.187 (0.283)	0.448 (***0.007***)	− 0.354 (0.037)^†^	0.086 (0.625)	− 0.272 (0.220)	0.608 (***0.003***)	− 0.735 (***< 0.001***)^†^	0.418 (0.053)

Bold-italicized numbers are statistically significant results with p < 0.01 (p value in parentheses). ^†^Correlation significantly different than counterpart group correlation *p* < 0.05 (one-tailed). Calculated only when at least one of the correlations compared is significant.

Higher propionate concentrations in the combined sample were associated with increased SOL and decreased SE. In the short group, higher propionate was significantly associated with increased SOL and decreased SE.

Higher concentrations of butyrate were associated with increased SOL and decreased TST and SE for the entire sample. Higher butyrate was associated with higher SOL and shorter SE in the short sleep duration group.

For the entire group, total SCFA concentration was associated with increased SOL and decreased SE. Higher total SCFAs was associated with higher SOL in both groups, short and normal sleep duration, and shorter SE in the former.

Overall, correlations were stronger in the short group than in the normal group. No significant correlations were found between isobutyrate, isovalerate, or valerate and any of the sleep continuity measures.

### Regression results

We performed multiple linear regression analysis to predict total SCFA concentrations by age, BMI, TST, SOL, and WASO (Table 3). The model was significant and explained 40.7% of the variance in total SCFA. Higher BMI, shorter TST and longer SOL significantly predicted higher fecal SCFA concentrations.

**Table 3:** Results of a multiple linear regression analysis predicting total SCFA concentration by age, BMI, TST, SOL, and SE.

Dependent Variable		Predictor	B	t	*p*	Semi-partial correlation	Explained variance (%)
Total SCFAs	*F*(5, 51) = 8.71, *p* < 0.001 Adjusted R^2^ = 0.407	Constant	290.321	1.352	0.182		
		Age	− 0.842	− 0.324	0.747	− 0.03	0.11
		**BMI**	5.966	2.995	**0.004**	0.30	9.49
		**TST**	− 0.627	− 2.929	**0.005**	− 0.30	9.07
		**SOL**	3.449	3.389	**0.001**	0.34	12.15
		WASO	0.163	0.278	0.782	0.02	0.08

*p*-value in bold indicates a significant predictor.

## Discussion

Our main findings are that in older adults, insomnia symptoms with short sleep duration are associated with significantly higher fecal SCFA concentrations than insomnia symptoms with normal sleep duration, and that these metabolites (specifically acetate, propionate, and butyrate) are significantly correlated with poorer sleep continuity outcomes, above and beyond the effect of BMI. Overall, these findings support our study hypothesis. Specifically, higher levels of acetate, butyrate, propionate and total SCFAs were significantly associated with lower SE and longer SOL, and higher butyrate levels were significantly associated with shorter TST in the entire sample. All associations with SE and SOL remained significant in the short sleep duration group, but not in the normal sleep duration group (except for acetate and total SCFAs and SOL). The regression model showed that total SCFAs were significantly predicted by TST, SOL and BMI. No associations were found with WASO. Levels of isobutyrate, isovalerate, and valerate were similar in both groups and were not correlated with sleep continuity measures.

Studies examining the role of SCFAs in sleep disturbance in animals and humans are scarce and inconsistent^27,28,36,46,47^. Nevertheless, evidence indicates that SCFAs may serve as key mediators in a range of neurological conditions via microbiota–gut–brain communication^15^. One plausible pathway linking SCFAs and sleep disturbance may be related to their binding to specific receptors in gut Enterochromaffin cells (ECCs), promoting serotonin synthesis^48^. Serotonin, a key neurotransmitter involved in sleep and mood, affects the brain through ECCs interaction with afferent nerve fibers through synapse-like connections between neuropod-like extensions and afferent nerve terminals^48^.

Another plausible pathway linking SCFAs and sleep disturbances may be related to another main neurotransmitter, noradrenaline. The SCFA propionate activates the Free Fatty Acid Transporter 3 (FFAR3), which is abundantly expressed in sympathetic ganglia^49^. Propionate-induced FFAR3 directly promotes noradrenaline release^50,51^, which is associated with increases in arousal^52^. Indeed, insomnia patients showed increased nocturnal levels of circulating noradrenaline, which was negatively correlated with sleep efficiency^53^. Furthermore, physiological hyperarousal has been implicated as a mechanism explaining the increased risk of hypertension in insomnia with objective short sleep duration^54,55^.

Our findings show that BMI average in the short sleep duration group was significantly higher than in the normal sleep duration group. These results are consistent with cross-sectional and prospective studies, demonstrating an association between sleep duration and body mass index (BMI) in both children and adults^56–59^. Higher BMI in the short sleep duration group may also indicate unidentified sleep apnea^60^. Indeed, both age and obesity are significant risk factors for sleep apnea^61^. Furthermore, the association between elevated fecal SCFAs and obesity has been demonstrated in human studies^62,63^. Higher SCFA excretion has been associated with gut microbiome dysbiosis, obesity, hypertension, and cardiometabolic disease risk factors^17^. These findings suggest that SCFAs may play a mediating role in the association between sleep and obesity. High levels of fecal SCFAs observed in older adults with high BMI and insomnia symptoms may be explained by low SCFA absorption in epithelial cells in the gut and chronic inflammation. These mechanisms warrant further investigation.

This study has potential limitations. Diagnosis of insomnia focused on nighttime symptoms but did not include assessment of daytime functioning; thus it does not meet full criteria for insomnia disorder, thereby limiting generalizability of the findings. However, it has been shown that the negative impact of nighttime sleep problems on daytime functioning declines with age^64^, suggesting that older adults learn to adapt to their poor sleep condition and experience less of an impact on their daytime functioning. As this investigation was part of a major study on insomnia symptoms in older adults, we could not compare SCFA concentrations with a non-insomnia control group. In addition, the study findings cannot be generalized to younger adults, since aging may play a role in SCFA concentrations. Moreover, as this was a cross sectional investigation, causality cannot be inferred. Inflammation parameters, which would allow a better understanding of the mechanisms underlying the observed associations between SCFA concentrations and sleep disturbances, were not assessed. Finally, volunteers were excluded if they reported sleep apnea and/or PLMS, yet these sleep disorders are underdiagnosed and thus we cannot rule out their presence in the study sample.

In summary, our results show that short sleep duration in insomnia is associated with an increase in SCFA concentrations in feces. These results are consistent with the idea that severe insomnia in older adults is accompanied by a chronic inflammation state, which may affect epithelial cells in the gut and decreased SCFA uptake. Since SCFAs are essential to gut cell function and brain signaling via neurotransmitters such as serotonin, reduction of SCFA absorption by the gut cells is expected to negatively affect sleep duration and continuity. Further research is needed to explore this theoretical framework.

Moreover, as SCFAs are products of dietary fiber fermentation in the gut, the development of dietary interventions based on specific fibers, aimed to ameliorate insomnia and other neurobiological diseases, may prove to be an interesting and rewarding area of research.

## Data Availability

The raw data are available from the corresponding author upon request.
